# Insights into the binding of GABA to the insect RDL receptor from atomistic simulations: a comparison of models

**DOI:** 10.1007/s10822-013-9704-0

**Published:** 2014-01-18

**Authors:** Federico Comitani, Netta Cohen, Jamie Ashby, Dominic Botten, Sarah C. R. Lummis, Carla Molteni

**Affiliations:** 1 Physics Department, King’s College London, Strand, London, WC2R 2LS UK; 2 Department of Biochemistry, University of Cambridge, Tennis Court Road, Cambridge, CB2 1QW UK

**Keywords:** Pentameric ligand-gated ion channels, GABA neuroreceptors, RDL receptor, Insecticide resistance, Molecular dynamics, Homology modelling, Ligand–protein docking, Metadynamics

## Abstract

The resistance to dieldrin (RDL) receptor is an insect pentameric ligand-gated ion channel (pLGIC). It is activated by the neurotransmitter γ-aminobutyric acid (GABA) binding to its extracellular domain; hence elucidating the atomistic details of this interaction is important for understanding how the RDL receptor functions. As no high resolution structures are currently available, we built homology models of the extracellular domain of the RDL receptor using different templates, including the widely used acetylcholine binding protein and two pLGICs, the *Erwinia Chrysanthemi* ligand-gated ion channel (ELIC) and the more recently resolved GluCl. We then docked GABA into the selected three dimensional structures, which we used as starting points for classical molecular dynamics simulations. This allowed us to analyze in detail the behavior of GABA in the binding sites, including the hydrogen bond and cation-π interaction networks it formed, the conformers it visited and the possible role of water molecules in mediating the interactions; we also estimated the binding free energies. The models were all stable and showed common features, including interactions consistent with experimental data and similar to other pLGICs; differences could be attributed to the quality of the models, which increases with increasing sequence identity, and the use of a pLGIC template. We supplemented the molecular dynamics information with metadynamics, a rare event method, by exploring the free energy landscape of GABA binding to the RDL receptor. Overall, we show that the GluCl template provided the best models. GABA forming direct salt-bridges with Arg211 and Glu204, and cation-π interactions with an aromatic cage including Tyr109, Phe206 and Tyr254, represents a favorable binding arrangement, and the interaction with Glu204 can also be mediated by a water molecule.

## Introduction

Pentameric ligand-gated ion channels (pLGICs) mediate fast synaptic transmission in the central and peripheral nervous system and are present in a variety of organisms [1–3]. They are the site of action of many drugs that treat a range of neuronal disorders such as Alzheimer’s and Parkinson’s diseases and are therefore major therapeutical targets; in invertebrates they are the target of insecticides [4–6]. pLGICs consist of five subunits arranged around an ion permeable pore; each subunit includes an extracellular domain (ECD) and a transmembrane domain (TMD) surrounding the pore. The binding of specific neurotransmitters to the extracellular domain stimulates the opening (gating) of the channel in the TMD, which allows ions to flow across the membrane modifying the cell activity [7].

pLGICs are complex transmembrane proteins, difficult to crystallize. Hence high resolution experimental structural information is very limited, which in turn hinders our detailed understanding of how pLGICs function. However, the recent availability of a few structures for complete pLGICs at atomic resolution are opening new and exciting avenues for understanding the atomistic details of the channels. These are the structures of the bacterial *Erwinia Chrysanthemi* ligand-gated ion channel (ELIC) [8, 9] and *Gloeobacter Violaceus* ligand-gated ion channel (GLIC) [10, 11] likely in closed and open states respectively, and of a eukaryotic glutamate-activated chloride pLGIC (GluCl) [12] from the nematode *Caenorhabditis Elegans*. Previously, only a medium resolution electron microscopy structure of the nicotinic acetylcholine receptor (nAChR, from the electric ray *Torpedo Californica*) was available [13]. In fact, most structural information for the pLGIC ECDs has been inferred by homology with the acetylcholine binding protein (AChBP) [14], a globular snail protein homologous to the ECD of the nAChR. Interestingly, all known pLGICs show remarkable structural similarities and conserved residues important for receptor function [2, 3]; hence the available structures of the above mentioned systems can be used to infer information about other pLGICs.

In this work we have investigated the invertebrate resistance to dieldrin (RDL) receptor, an anion-selective receptor gated by γ-aminobutyric acid (GABA), which is important for inhibitory neurotransmission. The RDL receptor is a target for insecticides and also plays a role in olfactory learning [15]. The GABA binding site in its extracellular domain has been poorly studied compared to vertebrate GABA_A_ receptor binding sites and is the focus of our computational investigation. While many insecticides do not act at this binding site, evidence from Cys-loop receptors has indicated that compounds binding here are likely to bind with higher affinity and selectivity. Thus, a better understanding in atomistic detail of this site could lead to the design of more effective compounds. Resistance to dieldrin, which gives the name to the RDL receptor, is linked with a mutation in the M2 helix and has not been addressed in this work.

A three dimensional experimental structure of the RDL receptor is not available. To elucidate the details of neurotransmitter binding, identify the binding site within the receptor and characterize the main neurotransmitter-receptor interactions, we have used homology modelling, ligand–protein docking and molecular dynamics (MD) simulations. MD simulations provide information on the stability of the homology models and of GABA binding to the receptor, on the dynamical bond network that GABA forms inside the receptor, on the conformational flexibility of the interacting GABA and on the role of water molecules that may mediate the ligand-receptor interactions that cannot be obtained by homology modelling and docking alone. The resulting data integrate, confirm and expand the available information from mutagenesis and electrophysiology experiments [16–18], and suggest new experiments related to residues identified in the calculations as potentially important for binding. They contribute to the construction of a reliable atomistic picture for the RDL receptor in a case where atomistic details cannot yet be experimentally resolved.

Building homology models is not a unique procedure and strongly depends on the choice of template and alignment. Hence, we built a series of homology models of the RDL receptor on different templates/alignments and assessed their behaviour, including differences and similarities, upon GABA binding. Ligand–protein docking techniques also show certain degrees of arbitrariness; they may influence the MD simulations that made use of their results as initial structures, given the limitation of computationally affordable time scales. In this context, techniques to accelerate rare events can be very useful, so we tested the use of metadynamics [19] to explore the free energy landscape of GABA binding to the RDL receptor as a function of selected conserved characteristics and complement the MD data.

The comparison of the models and their validation with experimental data allowed us to select a reliable model for the GABA bound RDL receptor that will be useful for future studies.

## Methods

For the homology models of the extracellular domain, we selected three experimentally resolved pentameric structures to be used as templates. Homology modelling techniques are based on the fact that the tertiary structure of biomolecules is more conserved than the amino acid sequence; hence a known protein structure can be used as a template to build the unknown structure of another protein that shares some degree of sequence identity. The quality of the model depends on the quality of the template, on the similarity with the template and on how this similarity is recognized through sequence alignment. A search carried out with FUGUE [20], a server specialized in alignment able to recognise homologue proteins by comparing sequences and structures, identified three “families” as classified in the Homstrad database [21] with a Z-score larger than 6.0, the threshold over which the server identifies homologous with 99 % of confidence. Their representative structures were the GluCl receptor 3RHW [12], the acetylcholine binding protein 1I9B [14] and the nicotinic acetylcholine receptor structure 2BG9 at 4 Å resolution obtained by electron microscopy [13]: the respective Z-scores were 50.1, 17.9 and 11.0. The Homstrad online database organizes in the same Homstrad “family” proteins that share sequence/structure similarity. We selected three templates representative of these families which are all homo-pentameric structures resolved by X-ray spectroscopy, specifically: (1) the ECD of GluCl from the nematode *Caenorhabditis Elegans* at 3.3 Å resolution 3RIF, which is the same as 3RHW but with the ligand glutamate [12], (2) the AChBP structure from the snail *Lymnaea Stagnalis* at 2.7 Å resolution 1I9B [14]; (3) the ECD of ELIC from the bacterium *Erwinia Chrysanthemi* at 3.3 Å resolution 2VL0 [8], which is an X-ray structure with a better resolution than the electron microscopy structure 2BG9. With respect to other acetylcholine binding protein structures of better resolution like 2Y7Y [22] and 4AFH [23], 1I9B has a higher sequence identity with RDL (20 % with respect to 19 and 13 % in alignments with FUGUE). While all the three selected templates are reasonable, none is perfect. In fact, GluCl is likely to be the most suitable template, but it has been co-crystallized with glutamate, ivermectin and Fab fragments [12]. Hence its structure may have been unnaturally affected by the presence of bulky ligands. The AChBP structure has the best resolution with respect to the pLGIC templates; however, it is a globular protein (evolved to bind a ligand), not an ion channel (evolved to undergo substantial conformational changes upon ligand binding). The ECD of ELIC does not have the Cys-loop, a structural motif consisting of a loop formed by a disulfide bond between two cysteine (Cys) residues separated by 13 amino acids, which characterizes the RDL receptor and other pLGICs. An X-ray structure at 3.9 Å resolution of ELIC in complex with GABA and flurazepam (PDB ID: 2YOE) was recently released [9], after we had built the RDL model using 2VL0 as template. While 2YOE, as a bound version of the protein, might have been potentially a better template, both sequence and tertiary structure are very similar for the two ELIC structures. 2YOE has an extra glycine in each subunit of the ECD, which would not increase the percentage of sequence identity, and the relative root mean square displacement (RMSD) of the backbone atoms is 1.3 Å, well below the experimental resolution. Most importantly for our study the binding pocket for GABA (consisting of the 7 residues that map into the binding pocket in the RDL homology models) are very similarly positioned, with an RMSD, including all atoms, of about 0.7 Å. Hence using 2YOE instead of 2VL0 as template would not improve the results.

In this study we chose to use templates with different characteristics, align them individually to the RDL sequence, and then compare the resulting models. An alternative approach based on multiple templates, which did not include GluCl, has been proposed and tested for the vertebrate GABA_A_ receptor [24]. The α_1_β_2_γ_2_ GABA_A_ receptor has recently been modelled using a chimera of GluCl and ELIC [25].

**Fig. 1:**
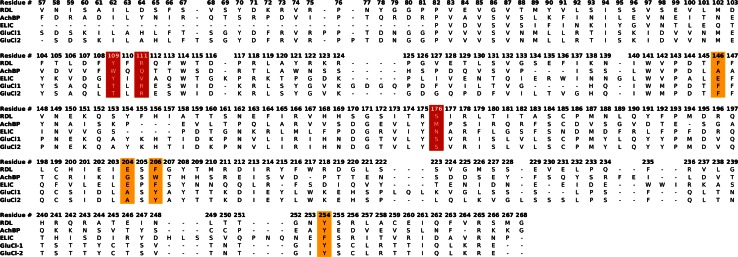
Sequence alignments of RDL with AChBP (~20 % sequence identity), ELIC (~24 %) and GluCl (~39 % for GluCl1 and ~38 % for GluCl2). The residues studied by mutagenesis experiments [17, 18] are highlighted (principal subunit: Phe146, Glu204, Phe206 and Tyr254; complementary subunit: Tyr109; Arg111 and Ser176)

The RDL receptor sequence used is for *Drosophila Melanogaster* (NCBI accession: NM_168321.1, residues 57-266). RDL receptor subunits can occur as different splice variants, which have minor changes in their D and F loops. The ‘ac’ variant used in this study is considered the canonical isoform [26]. The sequence identity with RDL is substantially larger for GluCl than for AChBP and ELIC. Because of this, the models produced with the GluCl template are likely to be the best. Hence we tested two different alignments produced with CLUSTAL [27] and FUGUE [20] (indicated as GluCl1 and GluCl2), at 39 and 38 % of sequence identity respectively. The sequence identity was low for AChBP and ELIC, at ~20 and ~24 % respectively; this made the alignments more difficult and questionable. Because the work is centred on GABA binding, particular attention was given to reproduce realistic binding sites for which there is experimental evidence [16–18]. We tested alignments obtained with various softwares and had to introduce small manual adjustments to position the residues of the binding site, in particular within the critical C loop, so to be structurally aligned with those of the templates. We then selected the alignments producing the best models, which were based on alignments obtained with FUGUE [20] for AChBP and with Modeller [28] for ELIC. Although the models built with these alignments are expected to be of a worse quality with respect to those built with the GluCl template, it is interesting to study and compare them. Other alignments (with small variations) could have also been considered for these low sequence identity templates to account, for example, for unstructured loops structural similarities in the templates; however, while they might marginally improve the resulting models, the overall quality would be similar to those presented here, especially when compared to the models obtained with the GluCl template. The selected alignments are shown in Fig. 1, where the residues studied by mutagenesis experiments [17, 18] are highlighted. As particular attention was given to reproduce realistic binding sites, the alignment of other parts of the protein, such as the variable region between the β8 and β9 strands, may be less accurate.

**Fig. 2:**
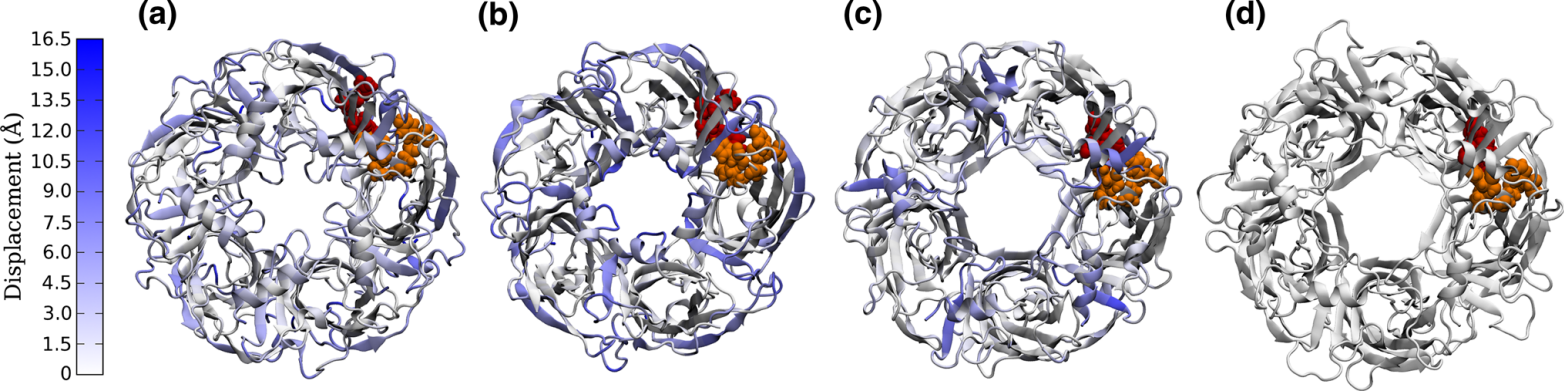
The selected homology models for the pentameric extracellular domain of the RDL receptor, after structure optimization: **a** RDL-AChBP; **b** RDL-ELIC; **c** RDL-GluCl1; **d** RDL-GluCl2. The seven residues Phe146, Glu204, Phe206 and Tyr254 in the principal subunit and Tyr109, Arg111 and Ser176 in the complementary subunit are explicitly shown. The displacement of the protein backbone from that of the RDL-GluCl2 model is highlighted

Homology models were built with the MODELLER 9.8 software package [28], imposing the disulphide bridges to reproduce the Cys-loop structural motif. Except for the model corresponding to the GluCl2 alignment, which was built as described in Ref. [18], the C_α_ arrangement was identically replicated for the five subunits, producing equivalent interfaces. Not imposing symmetry in GluCl2 did not result in any appreciable difference between subunits because of the very strong similarities of the subunits in the template. One hundred models were built with each template and evaluated according to the quality indicators GA341 and normalised Discrete Optimized Protein Energy (DOPE) scores [29–31]. The GA341 quality indicator combines a Z-score calculated for the combined statistical potential energy of a model (including solvent accessibility and distance-dependent terms), target-template sequence identity and a measure of structural compactness. The GA341 score ranges from 0 to 1, where 0 is for incorrectly folded models and 1 for models that should be comparable to at least low resolution X-ray structures [32]. For the best model selection, priority was given to the normalised DOPE score, but when in doubt among models with similar and non optimal DOPE scores (as was the case of ELIC), the model with GA341 closer to 1 was chosen. To further assess the reliability of the selected models and confirm the absence of severe structural problems, additional analysis was carried out with tools from the SWISS MODEL server [33], in particular QMEAN, which is a scoring function accounting for five different structure descriptors to consider the major geometrical aspects of a protein backbone [34]. Ramachandran plots were also evaluated with PROCHECK [35] to exclude any sizeable presence of residues in non-allowed regions. For each sequence alignment, the best model according to the criteria described was chosen for the GABA binding studies. The selected models are referred to as RDL-AChBP, RDL-ELIC, RDL-GluCl1 and RDL-GluCl2, which was also studied with a different protocol in Ref. [18]. Standard amino acid protonation at neutral pH was used. To eliminate any atomic clash, the models were optimized, after having been solvated with a 12 Å buffer of TIP3P water with a Na^+^ Cl^−^ saline concentration of 0.15 M and counterions to neutralize the structures, with the AMBER ff03 force field [36] within the AMBER 11 package [37]. The ff03 force field is widely used for biomolecular simulations and we had previously successfully used it for studying the binding of GABA to the GABA_C_ receptor, another pLGIC [38]. The use of other recently proposed force fields within the AMBER family, which improve on some of the limitations of ff03, is unlikely to significantly affect the results for the RDL extracellular domain, as improvements are mostly related to helical propensity which is of limited relevance to the RDL receptor ECD [39–42]. The solvated structures were then optimized in stages, progressively releasing restraints; each stage used first the steepest descent method and then the conjugate gradient algorithm. First the whole protein was restrained, in order to let the water molecules and the ions relax, then only the C_α_ of the protein backbone were restrained, to allow the optimization of the side chain; finally a structural optimization without restraints was performed. The four optimized pentameric models are shown in Fig. 2, where relative differences of the backbone, with respect to that of RDL-GluCl2, have been highlighted. Small variations in the five subunits of each model, especially in the unstructured loops, can be observed due to the random initial positions of the water molecules and ions; however, these variations were not big enough to make the five interfaces non-equivalent.

**Fig. 3:**
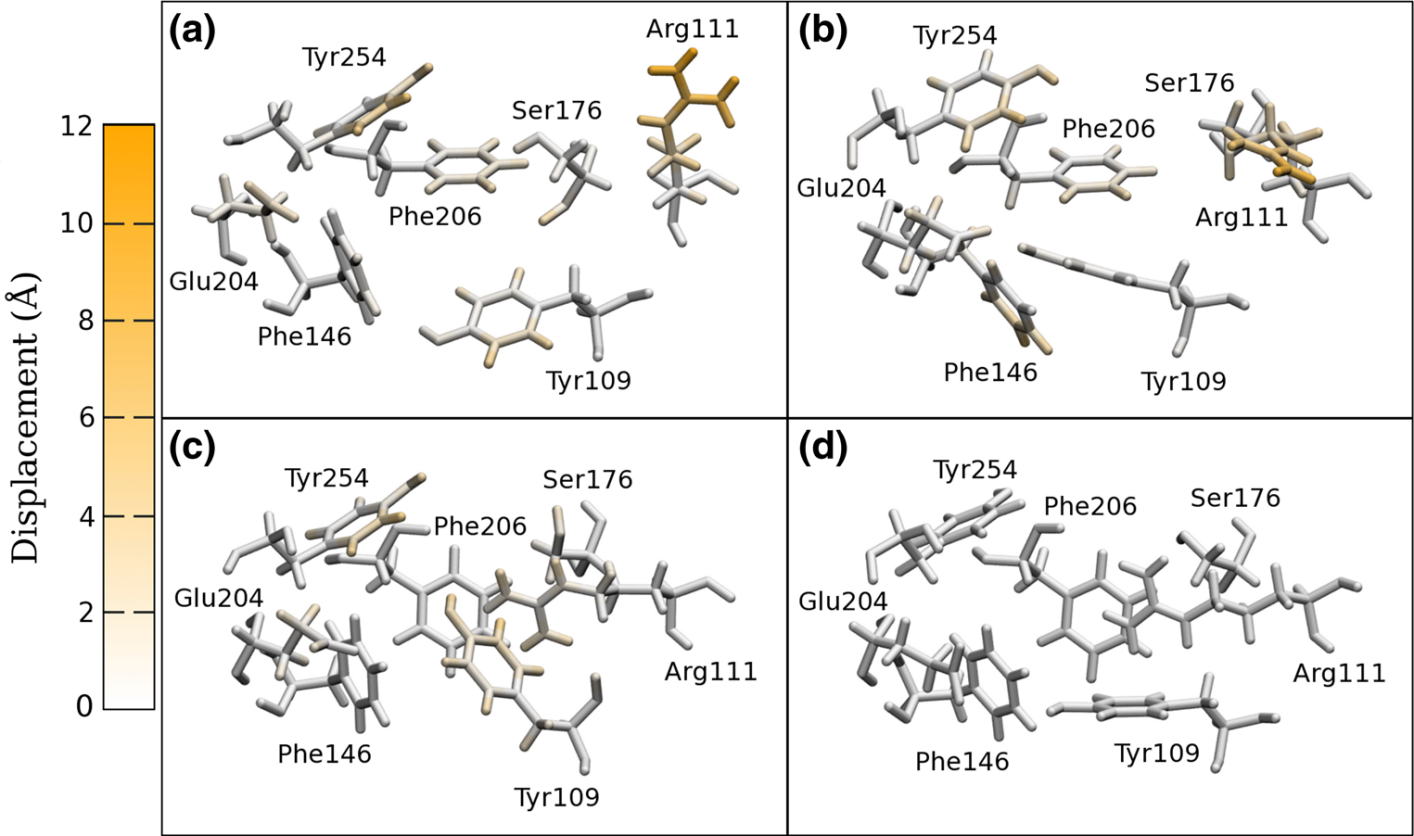
The GABA binding sites consisting of residues Phe146, Glu204, Phe206, Tyr254, Tyr109, Arg111 and Ser176, in the optimized homology models before GABA docking: **a** RDL-AChBP; **b** RDL-ELIC; **c** RDL-GluCl1; **d** RDL-GluCl2. The displacement of each atom from its position in the RDL-GluCl2 model is highlighted

Electrophysiology mutagenesis experiments have identified residues Arg111, Glu204, Tyr109, Tyr254 and Phe206 as important for neurotransmitter binding; Ser176 and Phe146 may also play a role [17, 18]. These seven amino acids (Phe146, Glu204, Phe206 and Tyr254 in the principal subunit and Tyr109, Arg111 and Ser176 in the complementary subunit) are highlighted in the models of Fig. 2, showing the location of one of the five equivalent binding sites in the pentameric structure and, on a larger scale, in Fig. 3, where the relative differences can be observed.

**Fig. 4:**
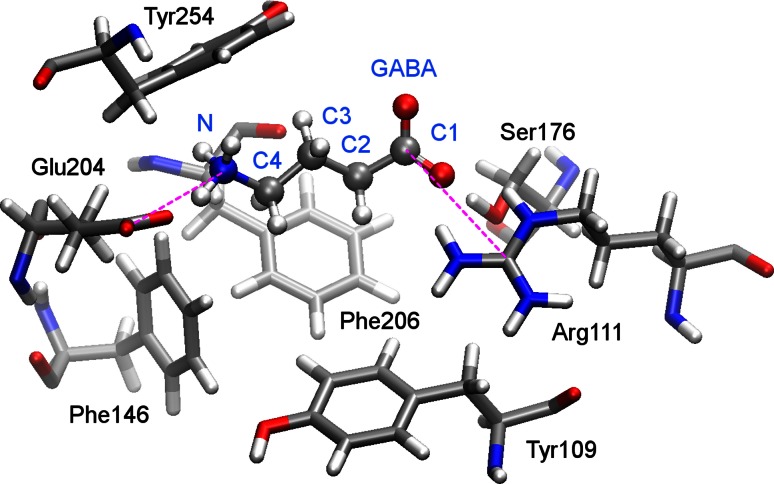
Example of GABA docked in the extracellular domain of the RDL receptor (RDL-GluCl1 model), with residues in the binding pocket. Labels to selected GABA atoms are shown. The *dashed lines* represent the collective variables used in the metadynamics simulation

GABA is believed to bind to the RDL receptor in its zwitterionic form, as shown in Fig. 4, which facilitates the interaction with charged amino acids in the binding site. As in other pLGICs such as the GABA_C_ receptor [38], interactions between the GABA negatively charged carboxylate group and an arginine residue, and between the positively charged amine group and aromatic residues such as tyrosine and phenylalanine are expected; this picture is consistent with the residues identified by the electrophysiology experiments.

GABA was docked into RDL-AChBP, RDL-ELIC and RDL-GluCl1, using the AUTODOCK 4.2 software [43], within a region containing Arg111, Glu204, Tyr109, Tyr254 and Phe206, whose side chains, as well as GABA, were flexible during the docking procedure. GABA was docked in RDL-GluCl2 within a 10 Å radius from Phe206 [18], with the docking software GOLD 4.0 [44]. Optimal activation of pLGICs requires the binding of at least two agonists likely in non-consecutive interfaces, but for the investigation of the binding network with MD simulations the interfaces can be considered independent; in fact some MD studies simulate only two subunits rather than the whole pentamer [45]. For simplicity, GABA was docked at one of the equivalent interfaces. The docking poses obtained were clustered according to their binding energy and conformational similarity, using a root mean square deviation of 2 Å as criterion. For each model the pose from the most populated cluster, with lowest energy and maximal potential interactions with the residues highlighted by experiments was chosen as a representative structure for MD simulations. As an example and to show relevant labels, the selected pose for GABA in the RDL-GluCl1 binding site is shown in Fig. 4; the models with one ligand will continue to be referred as RDL-AChBP, RDL-ELIC, RDL-GluCl1 and RDL-GluCl2. For further validation and to improve statistics, the docking poses were replicated at the five interfaces; the corresponding models will be indicated as RDL-AChBP-5, RDL-ELIC-5, RDL-GluCl1-5 and RDL-GluCl2-5.

The models of the RDL receptor ECD with either one or five docked GABA were then used as initial structures for MD simulations, using the AMBER ff03 force-field [36] and the AMBER11 package [37]. GABA partial charges were ESP partial charges (which reproduce the electrostatic potential) evaluated within density functional theory with the CPMD code [46], using a plane wave basis set with a kinetic energy cutoff of 70 Ry, Martins-Trouillier pseudopotentials [47] and the Perdew–Burke–Ernzerhof (PBE) exchange and correlation functional [48]. The partial charges were an average of the ESP charges for eight structures of GABA, in order to account, on average, for GABA flexibility during the MD. All the ECD structures were solvated with a 12 Å buffer of TIP3P water molecules in periodically repeated truncated octahedral cells. Na^+^ and Cl^−^ counterions were added for charge neutrality as well as for mimicking physiological conditions at a saline concentration of 0.15 M. The vibrational motion of bonds containing hydrogen atoms were constrained with the SHAKE algorithm [49], thus allowing a time step of 2 fs; all other bonds were allowed to vary in length. A cutoff of 10 Å was used for non-bonded interactions and the particle mesh Ewald method was employed for the long range electrostatic interactions. After minimization and thermalization, 25 ns of MD were carried out at 300 K, with a Langevin thermostat characterized by a collision frequency of 1 ps^−1^, and at 1 atm, with a Berendsen barostat with relaxation time of 2 ps. Restraints on the C_α_ were gradually removed in the first 3 ns, except for the five terminal amino acids of each subunits in order to mimic the presence of the transmembrane domain. The last 12 ns of MD, after all models had stabilized and equilibrated, were used for calculating statistical averages. Quantities calculated for the models with five ligands were averaged over the five binding sites.

Hydrogen bonds were evaluated using as criterion a donor–acceptor distance smaller than 3.5 Å and donor-H-acceptor angle larger than 120°. Cation-π interactions were characterized by a distance between the cation and the centre of the aromatic ring smaller than 6 Å [50] and by an angle between the normal to the ring and the direction joining the centre of the ring and the cation smaller than 45° [18, 38]. The conformations of GABA were monitored with the torsional angles ϑ and ψ, defined respectively by the atoms C1–C2–C3–C4 and C2–C3–C4–N, as labelled in Fig. 4.

The enthalpic contributions to the free energies of binding of GABA to the RDL receptor models were evaluated for the models with one ligand with the MM/PBSA and MM/GBSA methods as implemented in AMBER, which combine molecular mechanics energies with continuum solvation models within either the Poisson-Boltzmann Surface Area (PBSA) or the (less accurate but computationally cheaper) Generalized-Born Surface Area (GBSA) schemes [51–53]. For such calculations, snapshots in the MD trajectories of the models with one ligand were selected every 10 ps of MD and a salt concentration of 0.15 M was used. The entropic contributions were evaluated using normal mode analysis [51] on 12 representative snapshots, taken every ns. For computational economy, these calculations were performed for the two subunits at the interface of which GABA was bound; we verified that the enthalpic contributions to the GABA binding energy calculated with these two subunits were the same as those calculated with all five subunits.

Finally, exploratory metadynamics simulations were performed to sample the free energy surface (FES) of the binding site and overcome, to some extent, the time scale limitation of MD. Metadynamics is an efficient method for accelerating rare events and sampling the free energy of complex polyatomic systems [19]. It has been successfully applied for a variety of systems and processes in different fields, from condensed matter physics and materials science to chemistry and biophysics, [54] including the investigation of a potential molecular switch relevant for the gating of pLGICs [55] and other processes [56]. It can be very useful to elucidate details in binding mechanisms unattainable with docking and conventional MD [57, 58]. Metadynamics is based on a dimensional reduction, i.e. on the identification of a finite number of slowly varying collective variables (CVs) as a function of which it is possible to describe a given process and the underlying FES. The algorithm consists of a coarse-grained artificial (“meta”) dynamics, superimposed to the physical dynamics, performed in the space of the selected CVs, biased by a history-dependent potential, which is built as a sum of Gaussians, of appropriate height and width, centred along the trajectory of the CVs. The FES minima are progressively filled by these Gaussians, which discourage the system from revisiting regions already explored, and free energy barriers can be overcome through the saddle points. Eventually the FES is iteratively compensated by the sum of Gaussians, which provides a quantitative cast of the multidimensional FES.

The FES of the GABA binding site of RDL-GluCl1 was partially mapped as a function of two CVs, representing the distance between the carbon of the GABA carboxylate group and the carbon connected to the three nitrogens in the Arg111 side chain (CV_Arg_) and the distance between the nitrogen of the GABA amine group and the carbon of the Glu104 carboxylate group (CV_Glu_), as shown with dashed lines in Fig. 4. We used confining walls at 9 Å to avoid the sampling of regions too far away from the binding site. Metadynamics simulations were started from equilibrated MD structures, using the PLUMED 1.2 plugin [59] coupled to the AMBER 11 package with the same parameters and conditions as in the previous MD. The well-tempered version of metadynamics [60] was run at 300 K with a bias factor of 10. Gaussians with a 0.3 Å width and an initial height of 0.1 kcal/mol were deposited every ps for ~50 ns.

## Results

All selected homology models, shown in Fig. 2, were characterized by very high percentages of residues in allowed regions of the Ramachandran plot (specifically 99.4 % for RDL-AChBP, 98.7 % for RDL-ELIC, 98.5 % for EDL-GluCl-1 and 98.6 % for RDL-GluCl2). Structural quality indicators pointed to better quality for the models built on the GluCl templates (which have a similar value of QMEAN [34]) followed by that on AChBP and then ELIC. Structural differences between the models were quantified by the deviation of the backbone with respect to that of RDL-GluCl2 and are highlighted in Fig. 2.

The binding sites, defined by the seven residues studied experimentally Tyr109, Arg111, Phe146, Ser176, Glu204, Phe206 and Tyr254 [17, 18], of the minimized homology models before docking are shown in Fig. 3. Here the deviation of each atom of the residues of the binding site from its position in RDL-GluCl2 is highlighted in the four models. Despite the difference in the starting templates and alignment, the binding site is overall well conserved in all models. The main displacement is for Arg111. Both AChBP and ELIC do not have an arginine residue in the corresponding position in the alignment with the RDL receptor, at variance from GluCl. Hence, RDL-AChBP and RDL-ELIC might have more difficulties in representing realistically Arg111. For all the other residues, atoms tended to have a relatively small displacement from the reference models, confined to the side chains which were flexible in the docking procedure for Arg111, Glu204, Tyr109, Tyr254 and Phe206 and could also readjust during MD simulations. In particular Tyr109 was in the same rotameric state in the two GluCl based models, as verified with the MolProbity server [61], and behaved similarly during the corresponding MD simulations. Overall the binding sites show a good degree of similarity. The residues which make up the binding pocket of RDL-ELIC (as shown in Fig. 3) are in the positions equivalent to residues that constitute the binding pocket in the GABA bound ELIC structure 2YOE [9]. Hence, although we used 2VL0 rather than 2YOE as template for homology modelling with no a priori indication of where GABA would bind in ELIC, we obtained a model with a binding pocket homologous to that experimentally resolved by X-ray crystallography. This is an indirect validation of the alignment selected for the ELIC template.

**Fig. 5:**
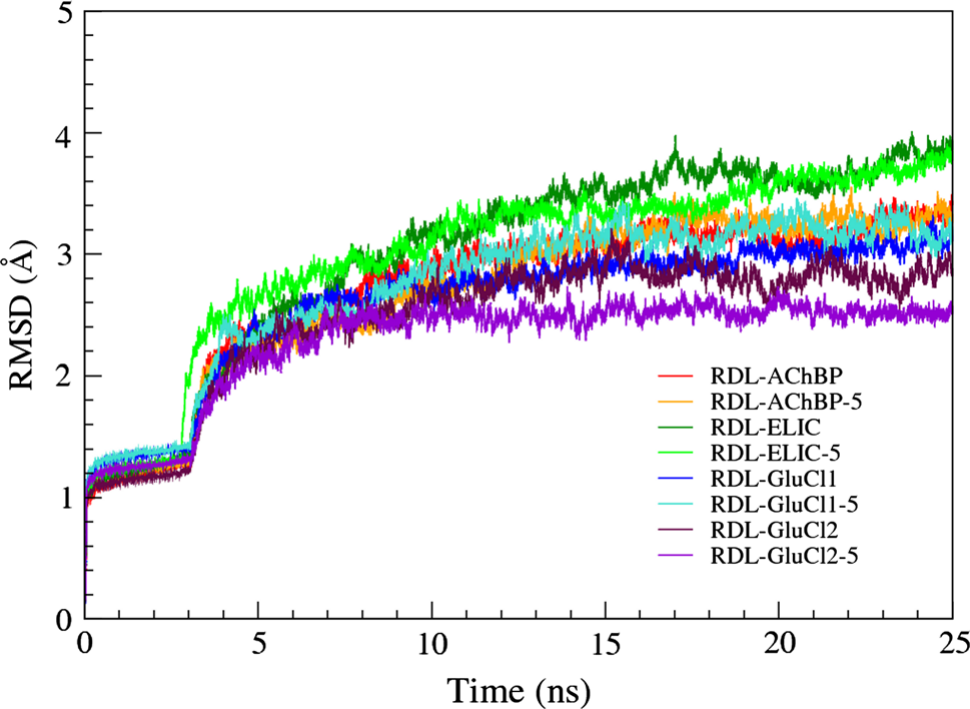
Root mean square displacements of the backbone atoms during the molecular dynamics simulations, including equilibration with the gradual release of restraints, for the RDL receptor models

The stability of the homology models was monitored throughout the MD simulations by the root mean square displacement of the backbone atoms calculated with respect to the initial optimized structure. The RMSDs for the MD simulations are shown in Fig. 5, during the equilibration procedures (including the release of all restraints but the last five residues of each subunit, which produced the jump at 3 ns) and the production runs. In the final 12 ns, when statistics were collected, the average RMSDs were 3.17 ± 0.11 Å for RDL-AChBP, 3.35 ± 0.09 Å for RDL-AChBP-5, 3.64 ± 0.13 Å for RDL-ELIC, 3.56 ± 0.16 Å for RDL-ELIC-5, 2.98 ± 0.09 Å for RDL-GluCl1, 3.19 ± 0.09 Å for RDL-GluCl1-5, 2.79 ± 0.12 Å for RDL-GluCl2 and 2.53 ± 0.06 Å for RDL-GluCl2-5. Statistical averages were calculated over the last 12 ns when all models were equilibrated and stabilized; RDL-ELIC and RDL-ELIC-5 were the slowest models to equilibrate. Average quantities evaluated over longer times (e.g. 18 ns for the RDL-GluCl2 models) or over the five binding sites did not change the overall picture. From the RMSDs, all models showed satisfactory stability, given their complexity, and GABA remained in the binding sites in all the MD simulations.

**Fig. 6:**
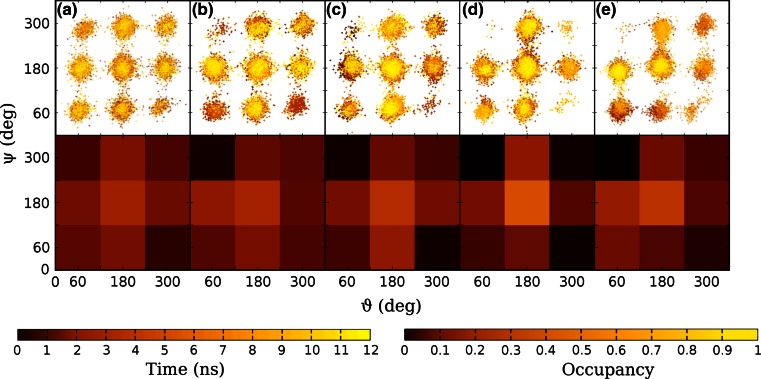
Time evolution (*top*) of the dihedral angles ψ and ϑ which characterize the GABA conformers, and conformer relative occupancy (*bottom*) during molecular dynamics simulations in **a** water, **b** RDL-AChBP-5, **c** RDL-ELIC-5, **d** RDL-GluCl1-5 and **e** RDL-GluCl2-5. The time evolutions of the five ligands are superimposed and the occupancies are averaged over the five binding sites

The conformations of GABA in its zwitterionic form inside the binding site can be characterized by the time evolution, shown in Fig. 6 for the models with 5 ligands, of the torsional angles ϑ and ψ defined in the previous section (top), and by the corresponding relative occupancies (bottom). Because of the force field, no hydrogen transfer between the GABA extremities is possible. The zwitterionic form should be favoured both in solution and in the receptor, due to its enhanced capability of interaction with solvent molecules and protein residues. There are 9 possible GABA conformers [62], characterized by values of ϑ and ψ around 60°, 180° and 300°. GABA is in the extended conformation when both ϑ and ψ are ~180° (centre of each panel in Fig. 6). It can potentially form intra-molecular hydrogen bonds between its carboxylate and amine group when in the conformers corresponding to the quadrants at the four corners (“corner” conformers), but in competition with inter-molecular bonds with either the solvent or the receptor. All conformers were easily accessible in water solution, with a preference for the extended configuration and low occupancies for the “corner” conformers. With respect to the case in water solution, as expected, in the RDL receptor models there were more restrictions for the conformers of GABA, with the top left conformers hardly populated. The extended conformer was overall preferred, without excluding the others: GABA can show some degree of flexibility and still stay bound to the RDL receptor models.

**Fig. 7:**
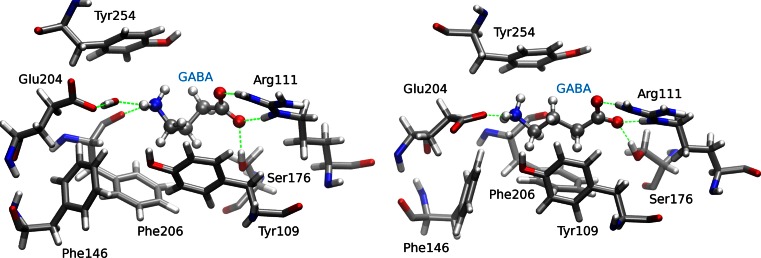
Molecular dynamics snapshots of GABA in the binding site of RDL-GluCl1 (*left*) and RDL-GluCl2 (*right*). Hydrogen bond interactions between GABA and the RDL receptor models are indicated with *dashed lines*

In Table 1 the average number of hydrogen bonds formed during the simulation time used to collect statistics is shown. The data for significant specific residues (i.e. forming at least 0.10 hydrogen bonds on average) are only shown and will be discussed for the models with five ligands, which have improved statistics. In all models bonding interaction with Arg111 was present. The positively charged Arg111 formed two or more salt bridges with the negatively charged carboxylate group of GABA in RDL-GluCl1-5 and RDL-GluCl2-5, while it formed on average less than one direct hydrogen bond in RDL-AChBP-5 and RDL-ELIC-5 where however it also participated in water-mediated hydrogen bonds. This discrepancy can be due to the positional difference of Arg111 in the RDL-AChBP model and RDL-ELIC models with respect to the models based on the GluCl templates, as evident in Fig. 4: AChBP and ELIC do not have an arginine in the corresponding position in the alignment. Interactions with Arg166 were also detected in RDL-AChBP-5, RDL-ELIC-5 and RDL-GluCl2-5; this interaction was also observed with low frequency in [18]. Glu204 formed salt bridges in all five models through its negatively charged carboxylate group interacting with the positively charged amine of GABA. An interaction here also occurred via a water molecule for significant percentages of the simulations. This is illustrated in Fig. 7, where snapshots from the MD simulation of RDL-GluCl1 (left, with a water mediated hydrogen bond between Glu204 and GABA) and RDL-GluCl2 (right, with a direct hydrogen bond between Glu204 and GABA) are shown. The polar residue Ser176 interacted with GABA through hydrogen bonds in all models consistently with experimental evidence; also Ser205 formed direct or water mediated hydrogen bonds. Thr250 interacted through hydrogen bonding with GABA in RDL-AChBP and in RDL-ELIC, but insignificantly in the other two models, where Thr251 formed hydrogen bonds. The aromatic residues Phe206, Tyr109 and Tyr254 formed fairly infrequent hydrogen bonds with GABA, the most populated of which occurred with Try254 in RDL-AChBP-5; they were however involved in cation-π interactions as described below. GABA formed more hydrogen bonds directly with the receptor in models built with the GluCl template, where more than five hydrogen bonds were observed in all cases. GABA also formed hydrogen bonds with water molecules in the binding site. The number of intra-molecular hydrogen bonds between the GABA carboxylate and the amine groups was very small in all cases, and linked to the occupancy of the “corner” conformers of Fig. 6. The total number of hydrogen bonds created by GABA in the MD simulations both with the receptor and water is lower than that found in water solution (~9.3 of which ~6 are with the carboxylate group and 3 with the amine group, exhausting all the possibilities to form hydrogen bonds).

**Table 1:** Average number of hydrogen bonds formed by GABA in the RDL-models and in water solution

	RDL-AChBP-5	RDL-ELIC-5	RDL-GluCl1-5	RDL-GluCl2-5	Water
**Arg111**	0.56 ± 0.67	0.71 ± 0.34	2.08 ± 0.65	2.16 ± 0.79	
H_2_O-mediated	0.46 ± 0.15	0.35 ± 0.20	0.17 ± 0.13		
Arg166		0.46 ± 0.50		0.55 ± 1.07	
H_2_O-mediated	0.28 ± 0.21	0.72 ± 0.56		0.17 ± 0.26	
**Glu204**	0.65 ± 0.34	0.44 ± 0.33	0.44 ± 0.36	0.60 ± 0.36	
H_2_O-mediated	0.49 ± 0.23	0.96 ± 0.11	0.61 ± 0.18	0.57 ± 0.40	
Glu246		0.17 ± 0.33			
**Phe206**	0.12 ± 0.15	0.12 ± 0.20	0.32 ± 0.24	0.22 ± 0.26	
**Ser176**	0.64 ± 0.44	0.58 ± 0.40	0.89 ± 0.19	0.94 ± 0.32	
H_2_O-mediated	0.20 ± 0.12	0.33 ± 0.15	0.14 ± 0.16	0.16 ± 0.19	
Ser205	0.62 ± 0.32	0.28 ± 0.33	0.57 ± 0.39	0.61 ± 0.30	
H_2_O-mediated	0.18 ± 0.13	0.31 ± 0.22	0.28 ± 0.30	0.24 ± 0.30	
Thr250	0.27 ± 0.47	0.33 ± 0.44			
Thr251			0.42 ± 0.37	0.53 ± 0.45	
**Tyr109**	0.54 ± 0.58	0.34 ± 0.35	0.27 ± 0.37		
**Tyr254**	0.12 ± 0.23	0.31 ± 0.39			
Protein	3.63 ± 0.67 (2.56)	3.80 ± 1.22 (4.23)	5.06 ± 0.62 (5.24)	5.72 ± 1.05 (6.79)	
H_2_O-mediated	1.88 ± 0.67 (2.82)	2.92 ± 0.51 (2.09)	1.53 ± 0.38 (1.05)	1.27 ± 0.96 (0.27)	
H_2_O	3.99 ± 0.59 (6.41)	4.32 ± 0.95 (3.77)	2.78 ± 0.68 (2.83)	1.97 ± 1.10 (0.27)	9.17
Intra-molecular	0.09 ± 0.13 (0.04)	0.02 ± 0.02 (0.05)	0.01 ± 0.02 (0.12)	0.04 ± 0.05 (0.00)	0.09
Total	7.71 ± 0.33 (9.01)	8.14 ± 0.30 (8.05)	7.85 ± 0.45 (8.19)	7.73 ± 0.26 (7.73)	9.28

The residues that were studied by mutagenesis electrophysiology experiments [17, 18] are in boldface. For interactions with specific residues, only average values equal or larger than 0.10 are shown; total values include all contributions. The data for the models with five ligands are averaged over the five binding sites; the data for the corresponding models with one ligand are shown in brackets

Data from many pLGICs have shown that positively charged groups in neurotransmitters sit within an aromatic cage [38, 63]. For example in the recent X-ray structure of ELIC in complex with GABA, the amino-moiety of GABA is caged by the aromatic side chains of Phe133, Tyr175, Phe188 and Tyr38, forming cation-π interaction with Phe133 and Phe188 [9].

The average number of cation-π interactions (Table 2) ranged from less than one on average in the AChBP based models, mostly due to Tyr254, to more than two in the GluCl based models, interacting with Phe206, Tyr109 and Tyr254 and also Phe164, with intermediate values for the models based on ELIC. No evidence of a direct interaction between GABA and Phe146 was found. This is consistent with mutagenesis data showing that a mutation of this residue with Ala produced only a moderate increase in EC_50_, indicating that there is no requirement for an aromatic residue in this position and that Phe146 does not contribute to cation-π interactions and is not critical for binding [17, 18]. The cation-π interactions of GABA with the aromatic residues kept the positively charged amine group mostly confined between them. This, together with the negatively charged carboxylate group pinned by the interaction with Arg111, favoured the extended conformation of GABA as shown in Fig. 6.

**Table 2:** Average number of cation-π interactions formed by GABA in the RDL models

	RDL-AChBP-5	RDL-ELIC-5	RDL-GluCl1-5	RDL-GluCl2-5
Phe164		0.12 ± 0.24	0.30 ± 0.33	0.13 ± 0.17
**Phe206**	0.12 ± 0.24	0.35 ± 0.34	0.64 ± 0.22	0.78 ± 0.24
**Tyr109**	0.05 ± 0.10	0.31 ± 0.35	0.22 ± 0.26	0.27 ± 0.23
**Tyr254**	0.80 ± 0.34	0.52 ± 0.34	0.97 ± 0.03	0.99 ± 0.01
Total	0.97 ± 0.15 (0.65)	1.33 ± 0.94 (1.53)	2.16 ± 0.68 (2.48)	2.18 ± 0.49 (2.46)

The residues that were studied by mutagenesis electrophysiology experiments [17, 18] are in boldface. The data for the models with five ligands are averaged over the five binding sites; the data for the corresponding models with one ligand are shown in brackets

The binding free energy for the models with one ligand evaluated within the MM/GBSA and MM/PBSA schemes are shown in Table 3, broken down into the enthalpic and entropic contributions. All water molecules were considered implicitly, although some mediated the interaction between the neurotransmitter and the receptor models, and so the results should be considered qualitative. MM/PBSA, which is more accurate but computationally more expensive, and MM/GBSA gave similar trends, consistent with the previous analysis of hydrogen bonds and the importance of cation-π interactions, with the weakest binding of GABA to RDL-AChBP (with significant contributions from water molecules in mediating ligand–protein interactions) and the strongest binding to RDL-GluCl2 (with minimal contributions from water and the extended conformer almost exclusively occupied). The differences between the binding energies of the two GluCl-based models with a single ligand are due to the different ratio between direct and water mediated interactions in the sampled configurations, which becomes similar by improving statistics in the models with five ligands.

**Table 3:** Enthalpic (ΔH) and entropic (TΔS) contributions and free energy of binding (ΔG) of GABA to the RDL receptor models with one ligand calculated with the MM/GBSA and MM/PBSA methods

Model	ΔH (kcal/mol) MM/GBSA	ΔH (kcal/mol) MM/PBSA	TΔS (kcal/mol)	ΔG (kcal/mol) MM/GBSA	ΔG (kcal/mol) MM/PBSA
RDL-AChBP	−8.3 ± 5.1	−7.2 ± 6.0	−14.5 ± 10.8	6.2 ± 12.0	7.3 ± 12.4
RDL-ELIC	−18.8 ± 3.2	−18.3 ± 5.1	−9.7 ± 5.5	−9.1 ± 6.3	−8.6 ± 7.4
RDL-GluCl1	−23.6 ± 3.8	−21.9 ± 6.4	−11.8 ± 6.5	−11.8 ± 7.5	−10.1 ± 9.1
RDL-GluCl2	−34.5 ± 3.0	−38.6 ± 4.0	−13.9 ± 7.6	−20.6 ± 8.1	−24.7 ± 8.6

**Fig. 8:**
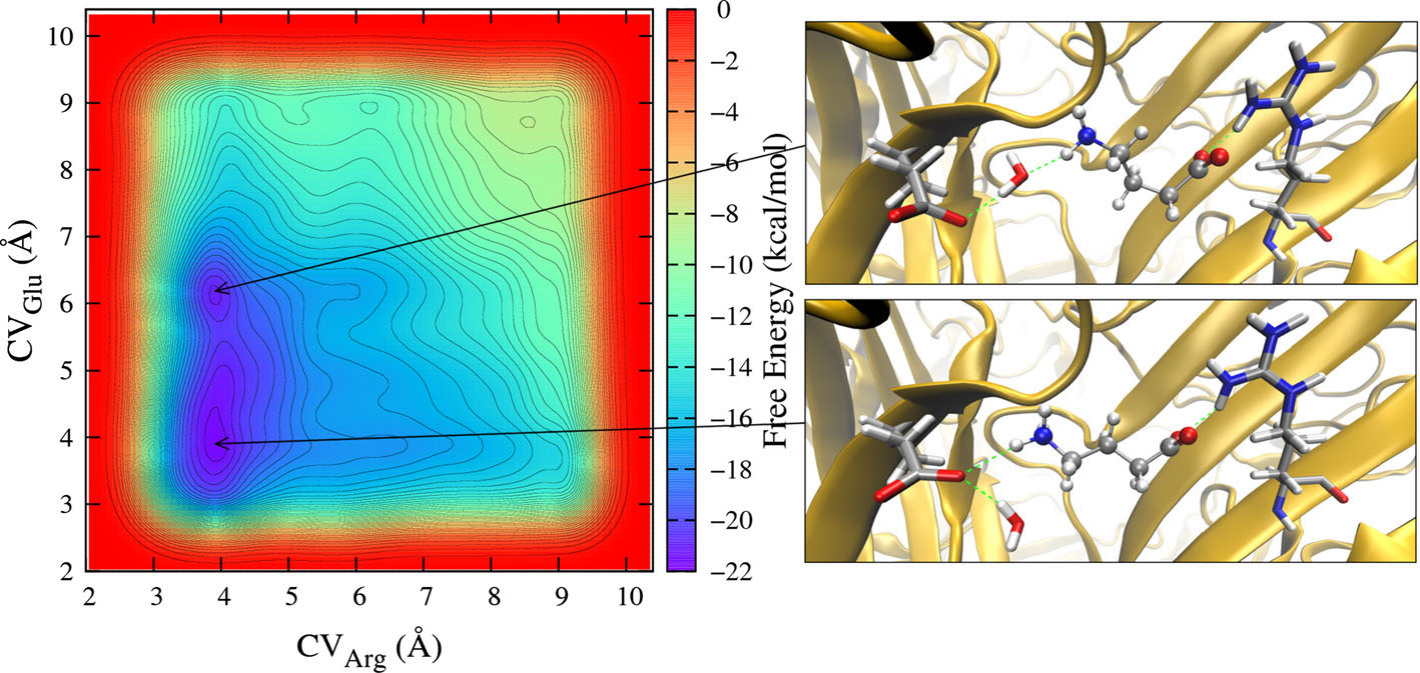
*Left*: Free energy map of GABA in the RDL-GluCl1 model as a function of the distance of the GABA amine from Glu204 side chain (CV_Glu_) and of the GABA carboxylate from the Arg111 side chain (CV_Arg_). *Right*: Binding arrangements corresponding to the free energy minima

The free energy landscape of GABA binding to the RDL receptor was explored by metadynamics as a function of the distance of the negatively charged carboxylate group of GABA from the side chain of Arg111 and that of the positively charged GABA amine from the side chain of Glu204, as detailed in the Methods section. Results for RDL-GluCl1 are reported in Fig. 8 as an example, showing an elongated basin of attraction corresponding to a range of relative distances between the amine and Glu204, while the GABA carboxylate was clearly pinned to Arg111. Two minima separated by a small barrier can be identified, related to the binding configurations shown on the right of Fig. 8: in one case (bottom) the amine group of GABA, which is in its extended conformer, is closer to Glu204 and forms a direct hydrogen bond; in the other case (top) GABA is in a bent conformation and forms a water mediated hydrogen bond.

## Discussion

All the models investigated were stable and GABA remained bound during the MD simulations, showing qualitatively common features in the binding pocket and in the interaction network of GABA. MD simulations are an excellent route to explore aspects of protein function that are difficult to probe by other methods, such as prolonged versus brief neurotransmitter-receptor interactions in the binding site, and details of specific bonds. Residues that have been identified experimentally as being important for binding played a clear role: salt bridge interactions with the positively charged Arg111 and the negatively charged carboxylate group of GABA, and between the positively charged amine group of GABA and the negatively charged side chain of Glu104 were always present, together with cation-π interactions of the GABA amine with aromatic residues. In addition other residues (e.g. Ser205 and Thr251) were suggested by the simulations as potential interaction partners and may be interesting to study experimentally. The data also indicated that water molecules play a role in mediating the neurotransmitter-receptor interaction, in particular when Glu204 was involved; water-mediated interactions have indeed been suggested for related systems, i.e. AChBP and possibly nAChR [64, 65].

Our data also illustrate the differences between the models. AChBP has been widely used as a template for Cys-loop receptor models, but it produced the least accurate of our models. RDL-AChBP was stable, but had fewer direct neurotransmitter-receptor interactions during the MD simulation and the weakest binding, with significant cation-π interactions only with Tyr254 at variance from other models and experimental findings. This is probably due to the model being based on a protein template with low sequence identity, which is likely to lead to inaccuracies; for example the important arginine of the binding site was not conserved. The models built on the GluCl template are of a better quality, showing more binding interactions and better overlap with experimental data, while RDL-ELIC is intermediate (ELIC has a relatively low sequence identity with RDL, but is a pLGIC). The RDL-ELIC model has on average more direct hydrogen bonds than RDL-AChBP and reasonable support from functional data; it also has more cation-π interactions than RDL-AChBP resulting in a stronger binding free energy. The trend is also maintained in the averages of the models with five ligands, although the differences between the AChBP and ELIC based models are less marked. These results show that both the percentage of sequence identity and the fact that the template is a pLGIC are important for obtaining reliable models. Comparison of MD simulations of RDL-GluCl2 and RDL-GluCl2-5 with the same model, similar docking recipe and multiple binding sites but a different protocol, reveals the same major interactions [18].

From the MD simulations we observed that different binding arrangements, e.g. in the simulations of GluCl based models, are not mutually exclusive, but may coexist or exist at different times and are consistent with the available experimental data. The proposal that there is a degree of flexibility in the binding arrangements is supported by our exploratory data on the binding of GABA to the RDL receptor obtained with metadynamics simulations, which identified two minima separated by a small barrier, related to the binding configurations in the presence and absence of a water molecule which mediates the interaction between the GABA amine and Glu204. The free energy landscape can be sampled as a function of other CVs and a search for the best representation is beyond the scope of the present paper, but these results show that metadynamics calculations can supplement MD simulations and experimental data providing useful insights to build a thorough picture of the binding of GABA to the RDL receptor.

The binding of GABA directly with Glu204 or through a water molecule is reflected by the conformation of GABA explored in the MD, with an extended conformation privileged for the direct salt bridge interactions. This is consistent with the analysis in [18], where the conformations of GABA were clustered according to the distance between the carbon of the carboxylate group and the nitrogen of the amine group. The most populated cluster (~80 %) was the one characterized by the largest distance between the carboxylate and the amine group, which would correspond to the maximally extended conformation in the centre of the central quadrant in Fig. 6. In the X-ray model 2YOE, [9] GABA is in an elongated conformation, characterized by the torsional angles *ϑ* ≃ 136^∘^ and *ϕ* ≃ 219^∘^, which would belong to the central quadrant of Fig. 6 corresponding to the extended conformation. One should bear in mind however that the resolution of the 2YOE structure is 3.9 Å and that GABA is a small molecule with a distance between the carbon atom of the carboxylate and the amine nitrogen of ~5 Å in the extended conformation. This conformer was also identified in other computational studies of GABA binding, e.g. in simulations of the GABA_C_ receptor [38] and of the α_1_β_2_γ_2_ GABA_A_ receptor [25]. In the latter study it was noticed that the GABA alkyl chain is fairly flexible, and hence unlikely to be entirely fixed in the protein bound state, and that even when not in a perfectly extended conformation GABA can find alternative optimal interactions in the binding pocket and bind equally well [25].

## Conclusions

We have here shown that a range of homology models, built on different templates and used for ligand–protein docking and MD simulations, were all stable and had broadly similar behaviour when studying the binding of GABA to the insect RDL receptor. There were differences, however, in specific details related to the interaction network, the role of water molecules and the conformation of the neurotransmitter. Comparing the various models from the different templates with experimental data revealed that the widely used AChBP template gave the least accurate results, while the GluCl receptor template produced the best models and more realistic simulations. We suggest that the latter can be used for future studies, which could for example exploit the power of metadynamics as a tool for exploring the binding mechanisms of the flexible GABA to the complex RDL receptor, as for this technique an accurate model is essential. Our preliminary studies indicate that metadynamics simulations could extend the information of MD simulations, providing data, for example, on the existence and relative occurrence of alternative binding or pre-binding poses, and the involvement and importance of water molecules.

## Abbreviations

pLGIC: Pentameric ligand-gated ion channel
ECD: Extracellular domain
TMD: Transmembrane domain
ELIC: *Erwinia Chrysanthemi* ligand-gated ion channel
GLIC: *Gloeobacter Violaceus* ligand-gated ion channel
GluCl: Glutamate-activated chloride ligand-gated ion channel
nAChR: Nicotinic acetylcholine receptor
AChBP: Acetylcholine binding protein
RDL: Resistance to dieldrin
GABA: γ-Amino butyric acid
FES: Free energy surface
MD: Molecular dynamics
PBE: Perdew–Burke–Ernzerhof
MM: Molecular mechanics
PBSA: Poisson–Boltzmann surface area
GBSA: Generalized Born surface area
CV: Collective variable
RMSD: Root mean square displacement
